# Heated Tobacco Products: Awareness, Beliefs, Use and Susceptibility among US Adult Current Tobacco Users, 2021

**DOI:** 10.3390/ijerph20032016

**Published:** 2023-01-21

**Authors:** Lindsey S. Sparrock, Lilianna Phan, Julia Chen-Sankey, Kiana Hacker, Aniruddh Ajith, Bambi Jewett, Kelvin Choi

**Affiliations:** 1 Department of Neuroscience, American University, Washington, DC 20016, USA; 2 Division of Intramural Research, National Institute on Minority Health and Health Disparities, Bethesda, MD 20892, USA; 3 Center for Tobacco Studies, Rutgers Biomedical and Health Sciences, New Brunswick, NJ 08901, USA; 4 School of Public Health, Rutgers Biomedical and Health Sciences, Piscataway, NJ 08854, USA; 5 School of Medicine, University of Pittsburgh, Pittsburgh, PA 15261, USA

**Keywords:** heated tobacco products, awareness, perceptions, tobacco use

## Abstract

Limited data exist on the awareness, beliefs, and use of heated tobacco products (HTPs). Data from 1583 U.S. adult (age ≥ 21 years) current tobacco users were collected in 2021. Participants self-reported HTP awareness, beliefs, use, and susceptibility, as well as current tobacco product use and sociodemographics. We used weighted logistic and multinomial regression models to explore their associations. Overall, 23.6% were aware of, 8.9% had ever used, and 3.0% currently used HTPs. Younger individuals (vs. 61+ years), those with annual income $50,000+ (vs. <$50,000), and those currently using electronic vaping products (vs. non-users) were more likely to be aware of, to have ever used, and to currently be using HTPs (*p* < 0.05). Black individuals (vs. White) were more likely to report ever and current HTP use (*p* < 0.05). Current cigarette smoking was not associated with HTP awareness and use (*p* > 0.05). Holding favorable HTP beliefs was associated with susceptibility to and more advanced HTP use statuses (*p* < 0.05). Sociodemographics associated with HTP use may reflect HTP marketing strategies. The lack of association with cigarette smoking suggests HTPs may be unlikely cigarette substitutes. Addressing favorable HTP-related beliefs may prevent dual use.

## 1. Introduction

Heated tobacco products (HTPs), also commonly referred to as “heat-not-burn” products by the tobacco industry [1], are electronic devices that heat a tobacco stick at lower levels of heating than required for ignition (approximately 350 degrees Celsius in heating vs. approximately 650 degrees Celsius in ignition) to generate an inhalable aerosol containing nicotine as well as other chemicals and toxicants [1,2,3,4]. Although there is a range of products available that use slightly different mechanisms to achieve the heating, they typically include a holder which is combined with processed tobacco in the form of tobacco sticks or capsules for use [5]. Currently, common HTP brands include *IQOS* (‘I-Quit-Ordinary-Smoking’; Philip Morris International [PMI]), *Eclipse* (R. J. Reynolds [RJR]), and *glo* (British American Tobacco [BAT]) [6].

A range of strategies have been used to promote HTPs, including the tobacco industry claiming they are safer than conventional cigarettes (e.g., [7,8,9,10]), opening dedicated stores using strategic pricing, and using various media platforms to promote and advertise HTPs [11,12]. As of 2021, HTPs account for approximately 3% of the global retail value of all tobacco products, nearly double the value seen in 2018 [6]. Based on the steady increase in sales, industry analysts predict that HTPs will represent approximately 30% of the total retail tobacco market and be valued at 77.61 billion USD by 2025 [13,14]. HTPs are currently available in many countries, with the largest markets being in Asia Pacific (approximately 73% market share in 2018), particularly Japan [15] and South Korea (e.g., [16,17]), as well as in Russia [6,14]. IQOS has continued to expand globally with test marketing occurring in over 50 countries as of May 2020 [18]. In the United States (US), HTP have been manufactured and commercially available since the 1980s but had not experienced widespread commercial success until recently [6,19]. On April 30, 2019, the US Food and Drug Administration (FDA) permitted the sale of IQOS through the premarket tobacco product application. Shortly thereafter, in October 2019, PMI introduced IQOS in Atlanta, Georgia [20] and by May 17, 2021, IQOS had expanded to Buford, Georgia; Richmond and Tysons, Virginia; North Charlotte and Raleigh, North Carolina; and Charleston and Myrtle Beach, South Carolina [21]. However, IQOS is currently unavailable for sale in the US due to an issued order for an ongoing patent infringement case filed by RJR [22]. Despite this dispute, PMI is likely to return IQOS to the US market in the near future and continue expanding in the US [21].

Public health officials have raised concerns over these products [16,23,24]. These include the dual use with other traditional tobacco products (e.g., cigarettes) and electronic cigarettes [25], the lack of evidence on the effectiveness of HTPs as a smoking quitting aid [26], and HTPs serving as a “gateway” for other tobacco product use by nicotine-naïve youth and young adults (e.g., [27,28]). Additionally, the safety and associated health risks of these products are also concerns [7]. In fact, although HTPs produce lower levels of some carcinogens and toxic chemicals in comparison to conventional cigarettes, they still pose some risks (e.g., [1,29,30]). Moreover, research has found that HTPs produce novel chemicals that are not generated by conventional cigarettes [7], further illustrating the uncertainty regarding the immediate and long-term HTP-related risks [29,31]. Additional evidence is slowly arising to fill in this gap [32,33]. Nonetheless, on July 7, 2020, the US FDA granted IQOS’s modified risk tobacco product (MRTP) application based on IQOS heating tobacco rather than burning it, as well as reduction of exposure to some harmful and/or potentially harmful chemicals [34]. Such modified risk claims may inadvertently mislead individuals to believe these products are “risk-free” [35].

Given the industry strategies and regulatory context outlined above, an evaluation of the prevalence of HTP use, awareness, and beliefs is essential for understanding its public health implications. However, there are a limited number of studies that have examined HTP use, awareness, and beliefs. A 2020 study analyzing data from 28 European countries found that 6.5% of respondents had ever used HTPs, 1.3% were currently using HTPs, and 0.7% were using HTPs daily [36]. In Asia, HTP awareness, use, and perceptions have also been examined with Japan having the highest prevalence of HTP use. In 2020, 10.9% of youth and adults (aged 15–74 years) were currently using HTPs with IQOS being the most commonly used brand (5.7%) [37]. A similar 2018 study in South Korea found that among tobacco users 36.8% had ever used HTPs, 28.3% were current users (past 30-day use), and 14.5% were co-using HTPs and other tobacco products [38]. Furthermore, HTP perceptions in Asian countries appear to be mostly positive. For instance, a cross-sectional analysis of South Korean adults found that current e-cigarette or HTP users (vs. non-e-cigarette or non-HTP users) perceived e-cigarettes or HTPs as less harmful than conventional cigarettes, and their positive perceptions were associated with greater use frequencies [39]. Similar findings were reported among South Korean tobacco users [38]. Additionally, focus group participants (aged 20–39 years) in Japan consistently reported IQOS as a “clean, chic and pure product” [40]. In the US, the awareness and use of these products appear to have rapidly increased in recent years. For example, a study of US adults between 2016 and 2017 found that within that year HTP awareness, ever use, and current use had increased from 9.3% to 12.4%, and 1.4% to 2.2%, and 0.5% to 1.1%, respectively [41]. More recent analyses of data from 2019 and 2020 found that between 8.1% and 15% of adults reported having heard of HTPs [42,43,44,45,46], 0.51–5% of adults reported ever using HTPs [42,43,44,45,46], and 0.10–2.4% of adults reported currently using HTPs [44,46].

More recent data are needed to track the awareness, beliefs, and use of HTPs, since the majority of the present research is from data collected between 2016 and 2019. Furthermore, the coronavirus (COVID-19) pandemic has been shown to have influenced tobacco use behaviors [47,48,49]. Additionally, data on commercial tobacco users, a group that could be at high risk of HTP use, are rare as only two studies to date have examined HTP use and awareness among a sample of current and former tobacco users (e.g., [50,51]), and no studies have examined HTP-related beliefs among this group. Therefore, the present study aimed to (1) estimate the prevalence of HTP awareness, use, and beliefs among US adult commercial tobacco users; (2) investigate the sociodemographics and tobacco product use associated with these measures; and (3) test the associations between various HTP-related beliefs and HTP use statuses to fill these research gaps. 

## 2. Methods 

### 2.1. Study Population

Data were from the COVID-19 and Commercial Tobacco Use Study (CaCTUS), which surveyed a nationally representative sample of U.S. adult recent former and current commercial tobacco users during January–February 2021 [52]. Eligible respondents included adults who were aged ≥ 21 years, currently using commercial tobacco and/or having used tobacco in the past 12 months (including cigarettes, cigars, e-cigarettes, hookah and other combustible tobacco products, and smokeless tobacco products), and residing in the US. Respondents were recruited from the YouGov online survey panel, which uses a sample-matching approach to achieve national representation. Briefly, YouGov randomly sampled individuals who met study inclusion criteria from existing national surveys to establish a reference sample. YouGov then recruited a sample of their panelists who met the inclusion criteria and matched sociodemographics to the reference sample through email invitations. Black and Asian adults were oversampled to allow for stable statistical estimates among these populations. Panelists went through further eligibility screening. Those eligible were invited to complete the online survey after providing informed consent and were compensated according to YouGov policy. Of the 2404 eligible panelists, 2123 completed the survey (cooperation rate = 88.3%). Survey responses were examined and respondents with inconsistent responses or in groups that exceeded the study quota were excluded, resulting in a final sample of 1700 respondents. Post-stratification weighting was used to achieve national representation. The current analysis was further restricted to current commercial tobacco users (n = 1583). This analysis did not require review or approval by the National Institutes of Health Institutional Review Board per 45 CFR 46 because it involved de-identified data and therefore is considered “not human subjects research”.

### 2.2. Measures 

#### 2.2.1. HTP Awareness, Use and Susceptibility

Respondents were presented with images of two popular brands of HTPs (IQOS and Glo) and asked the following questions: “Have you ever seen or heard of a heated tobacco product before this study?”. Those who answered “yes” were classified as being aware of HTPs, and those who answered “no” or “not sure” were classified as being unaware of HTPs. Respondents who were aware of HTPs were asked “Which of the following best describes your experience with a heated tobacco product?” (Response options: never used it before, used it before but not currently, currently using it some days or every day.) Respondents who were unaware of HTPs were classified as “never used it before”. Additionally, all respondents were asked: “Are you curious about heated tobacco products like IQOS?”, “Do you think you will try heated tobacco products like IQOS soon?”, “Do you think you will try heated tobacco products like IQOS in the next year?”, and “If one of your best friends were to offer you a heated tobacco product, would you use it?” (Response options: definitely not, probably not, probably yes, definitely yes). Respondents who answered “definitely not” for all four questions were classified as non-susceptible to HTP use. Otherwise, respondents were classified as susceptible to HTP use. Respondents were further categorized into four groups: non-susceptible never HTP users (non-susceptible and never used HTPs), susceptible never HTP users (susceptible but never used HTPs), ever-non-current HTP users (used HTP before but not at the time of the survey), and current HTP users (used HTPs some days or every day at the time of the survey). 

#### 2.2.2. HTP Beliefs

Given IQOS has the most market share of HTPs in the US, respondents were randomized into seeing one IQOS advertisement or a neutral image. They were then asked to rate their agreement, on a 4-point Likert scale, with the following HTPs (i.e., IQOS) perception statements: “IQOS is less harmful than cigarettes”, “IQOS is less addictive than cigarettes”, “IQOS is less harmful than other electronic vaping products (e.g., e-cigarettes)”, “IQOS is less addictive than other electronic vaping products (e.g., e-cigarettes)”, “Using IQOS occasionally does not cause any harm to the users”, “Using IQOS occasionally does not cause users to be addicted to nicotine”, “Using IQOS is not harmful to other people nearby”, “Using IQOS is socially acceptable”, and “The US Food and Drug Administration (FDA) approved IQOS as a safe product”. These statements were presented in random order to avoid an ordering effect. Responses were categorized as “agree” (including “strongly agree” and “somewhat agree”), “disagree” (including “strongly disagree” and “somewhat disagree”), and “don’t know”.

#### 2.2.3. Sociodemographic and Commercial Tobacco Use Statuses

Respondents also provided demographic information, including age, sex, race/ethnicity, educational attainment, annual household income, and urbanicity (see Table 1 for variable categories). Current use (i.e., currently using the product some days or every day) of cigarettes, electronic vaping products (EVP), cigars (including premium cigars, cigarillos, and little filtered cigars), hookah, other combustibles, and smokeless tobacco at the time of the survey were also assessed. 

### 2.3. Statistical Analyses 

Data were weighted to be representative of the US adult current and recent former commercial tobacco users. Sample characteristics (i.e., demographics, tobacco product use status) were summarized in weighted percentages. Prevalence estimates of awareness of HTPs and HTP use, as well as HTP-related beliefs overall and by sample characteristics, were calculated. Weighted logistic regression models were used to examine the association of demographics and tobacco product use status with HTP awareness, ever use, and current use. Specifically, the first set of models included only demographics, and the second set of models additionally included tobacco product use statuses. We employed this approach so that we did not dilute the association between demographics and HTP awareness and use since demographics can be causally related to HTP awareness and use through other tobacco product use. Finally, weighted multinomial logistic regression was conducted to examine the associations between HTP-related beliefs and HTP use statuses. Each belief was modeled separately, adjusting for demographics and tobacco product use statuses. Because beliefs did not differ significantly by the randomized images respondents saw, we did not include the image seen as a covariate in the model. All analyses were conducted in SAS^®^ Enterprise version 9.4 (SAS Institute, Inc.: Carey, NC, USA). 

## 3. Results 

### 3.1. Prevalence of HTP Awareness, Use, and Susceptibility

Table 1 summarizes the characteristics of the survey respondents after weighting. It also shows the prevalence of awareness, ever use, and current use of HTP by sample characteristics, as well as the adjusted odds ratios (AORs) between these characteristics and HTP awareness and use. Overall, 23.6% of US adult commercial tobacco users were aware of, 8.9% had ever used, and 3.0% currently used HTPs. Adjusting for all other factors, individuals aged 31–45 years (vs. 61+ years), Asians (vs. White), and current EVP users (vs. non-current users) were more likely to be aware of HTPs. Additionally, individuals between 18–45 years old (vs. 61+ years old), self-identified as Hispanic, Asian, Black, or other races (vs. White), reported current use of electronic vaping products (vs. non-current use), current use of hookah (vs. non-current use), and were more likely to have ever used HTPs. Individuals between 18–60 years (vs. 61 years or older), self-identified as Black or other races (vs. White), reported current use of electronic vaping products (vs. non-current use), hookah (vs. non-current use), and other combustibles (vs. non-current use), and were more likely to be currently using HTPs. In contrast, those making less than $50,000 per year (vs. $50,000 or more), and those living in either small cities or suburbs (vs. big cities) had lower odds of being aware of, having ever used, or currently using HTPs.

### 3.2. HTP Beliefs and Use Status

Table 2 shows the prevalence of various IQOS beliefs assessed in this study. Overall, 23.0% of these individuals agreed that IQOS is socially acceptable, 19.7% agreed that IQOS does not harm bystanders, 18.3% agreed that IQOS is less harmful than cigarettes, 16.3% agreed that using IQOS occasionally is not harmful, 15.4% agreed that IQOS is approved by the FDA to be safe, 15.0% agreed that IQOS is less addictive than cigarettes, 14.5% agreed that IQOS is less harmful than EVP, 13.5% agreed that IQOS is less addictive than EVP, and 13.5% agreed that using IQOS occasionally is not addictive. Additionally, Table 2 shows the relationships between IQOS beliefs and HTP use statuses. Overall, 29.9% of US adult commercial tobacco users were non-susceptible never HTP users, 61.3% were susceptible never HTP users, 5.9% were ever-not-current HTP users, and 3.0% were current HTP users. Across all HTP-related beliefs, a higher prevalence of agreement was associated with higher odds of more advanced HTP use statuses. For example, compared to those who disagreed that IQOS is approved by the FDA to be safe, those who agreed had higher odds of being susceptible never HTP users (AOR = 3.14, 95% CI = 1.42, 6.92), ever-not-current HTP users (AOR = 3.50, 95% CI = 1.30, 9.42), and current HTP users (AOR = 10.86, 95% CI = 3.05, 38.68) than non-susceptible never users. In contrast, reporting uncertainty about some of these beliefs was associated with lower odds of HTP use. For example, compared to those who disagreed that IQOS is approved by the FDA to be safe, those who reported “don’t know” had lower odds of being ever-not-current HTP users (AOR = 0.33, 95% CI = 0.14, 0.75). 

## 4. Discussion

This study provided updated estimates on the prevalence of HTP awareness, ever use, and current use among a nationally representative sample of US adult current commercial tobacco users. Overall, 23.6% of these adults reported awareness of HTPs, 8.9% reported ever use, and 3.0% reported current use in 2021. Our findings suggest that awareness of, experimentation with, and current use of HTPs remain stable since 2018. This may be due to the fact that soon after the US FDA authorized the distribution and marketing of IQOS as an MRTP [34] in 2020, IQOS was barred from manufacturing or selling IQOS in the US due to disputes with RJR in 2021 [22]. However, PMI intends to have IQOS available to the US market as early as 2023 [21]. This is likely to be accompanied by a substantial amount of marketing activities, which could potentially increase the awareness and use of these products. Furthermore, a major concern revolving around awareness of HTPs is the narrative pedaled by the tobacco industry that HTPs are safe, attractive, and healthier alternatives to conventionally smoked tobacco products (e.g., cigarettes) which in turn may mislead and entice current smokers and non-smokers to try out these products. The low prevalence of ever and current use of HTPs in this population may be due to potentially high initial monetary investment in the product. For instance, IQOS has a high start-up cost, with a device and pack of 200 HeatSticks selling for approximately $80 or more. However, some HTP marketers have started giving out free or low-cost samples [20], which may encourage more people (including nicotine-naïve individuals) to try these products and subsequently start using HTPs.

Our study is the first to examine HTP-related beliefs and their associations with HTP use statuses. Between 13.5% (IQOS is less addictive than electronic vaping products; using IQOS occasionally is not addictive) and 23.0% (IQOS is socially acceptable) of these individuals held favorable beliefs about IQOS, and perhaps HTPs in general. Holding these beliefs was associated with susceptibility to and more advanced/higher levels of HTP use. This is particularly concerning as many of these beliefs have not been validated by scientific evidence. It is important to point out that 15.4% of US adult current tobacco users believed that FDA-approved IQOS is safe. Although the FDA authorized IQOS as a reduced-exposure product, it did not authorize IQOS as a reduced-risk product, since reduced exposure does not inherently reduce the risks. Nonetheless, many individuals may have misinterpreted the authorization as an FDA-approved safe product. As such, it is important to effectively communicate what reduced exposure means to consumers and correct potential misbeliefs. 

Two correlates of HTP use are noteworthy. First, Black adults were more likely than White adults to have ever used and currently be using HTPs. Similarly, Nyman et al. (2018) found that Black US adults were more likely to be currently using HTPs than White adults in 2016 and 2017 [41]. Likewise, in Miller and colleagues’ 2020 study examining current and former tobacco users, minority ethnicity individuals (which includes Black individuals) were more likely to be aware of and have tried HTPs [51]. Since IQOS entered the US tobacco market, Philip Morris International (PMI) opened its first IQOS store in Atlanta, Georgia, a city that has a high proportion of Black/African American residents [53]. Additionally, many of IQOS advertising materials also featured Black models, and PMI markets and sells menthol-flavored IQOS [54] which are commonly used by Black/African American individuals (e.g., [55,56,57]). Our findings, together with those from previous studies, suggest that IQOS marketing strategies are potentially effective in targeting Black individuals to be aware of and use the product. Future studies assessing exposure to IQOS marketing activities and its awareness and use will provide evidence on how these activities may influence IQOS use. Second, current cigarette smoking was not associated with HTP use. This finding was supported by Nyman and colleagues (2018), who found that current HTP use was not associated with current cigarette smoking [41]. This is important because IQOS is authorized as a reduced-exposure product. For such reduced exposure to possibly translate to reduced risks, those who currently smoke cigarettes need to completely switch to HTPs. This is not supported by our findings because if cigarette smokers switch from cigarettes to HTPs, we would expect a higher prevalence of HTP use among former cigarette smokers, instead of a null association.

The present study is not without limitations. First, our sample lacks generalizability to nicotine-naïve adults and youth. However, current research suggests that initiation and maintenance of these products is lower among these populations. Nonetheless, public health officials may want to dedicate additional attention and efforts toward understanding HTP awareness and use, as well as related beliefs in these populations. Second, there is a potential for misclassification or discrepancies in how participants understood items, particularly among participants who were not familiar with HTPs prior to the survey. We tried to mitigate the risk of this issue by providing participants with the option to respond “don’t know” for HTP-related belief items. Third, respondents were surveyed in 2021 during the COVID-19 pandemic. It is unclear how the pandemic may have influenced participants’ beliefs (particularly in relation to health risks) and the use of these products. Fourth, given the low prevalence of current HTP use, some statistical comparisons may lack sufficient statistical power to detect a small to medium effect size. Therefore, continuing surveillance with a large sample size is needed to confirm our findings. Finally, the survey only specifically referred to IQOS when assessing HTP-related beliefs. Although IQOS is the most recognized HTP in the US, our findings on beliefs may or may not generalize to all HTPs, especially if they are marketed differently to consumers.

## 5. Conclusions

Overall, this study helps to enhance our existing knowledge of awareness and use prevalence, as well as beliefs about heated tobacco products among adult current commercial tobacco users in the United States. A notable proportion of these adults were aware of these products, but few reported currently using them. The favorable beliefs of HTPs were associated with more advanced/higher levels of heated tobacco product use. Together, these findings suggest a significant need for continued surveillance of HTP awareness, use, and related beliefs in an effort to gauge the need for public health interventions as well as regulations. 

## Figures and Tables

**Table 1:** Weighted prevalence of heated tobacco product awareness, ever use, and current use and their associations with sociodemographics and tobacco product use statuses, 2021 (n = 1583).

	Correlates	Overall %	Aware of HTP		Ever Used HTP		Current HTP Use	
			%	AOR (95% CI)	%	AOR (95% CI)	%	AOR (95% CI)
	**Age**							
	18–30 years	25.8%	24.6%	1.66 (0.86, 3.20)	11.2%	**3.10 (1.11, 8.72)**	2.2%	**21.05 (2.42, 183.01)**
	31–45 years	32.4%	27.6%	**1.93 (1.09, 3.39)**	12.3%	**3.72 (1.44, 9.64)**	6.2%	**54.34 (6.77, 437.12)**
	46–60 years	26.6%	22.4%	1.46 (0.78, 2.74)	5.9%	1.65 (0.55, 4.96)	1.4%	**10.48 (1.10, 100.25)**
	61+ years	15.2%	15.4%	Ref.	2.8%	Ref.	0.1%	Ref.
	**Sex**							
	Male	59.7%	29.0%	**2.15 (1.47, 3.14)**	10.6%	1.60 (0.97, 2.64)	3.1%	1.20 (0.54, 2.69)
	Female	40.3%	15.6%	Ref.	6.4%	Ref.	2.7%	Ref.
**Demographics**	**Race/Ethnicity**							
	Hispanic	13.2%	27.2%	1.36 (0.83, 2.25)	17.1%	**3.28 (1.61, 6.71)**	1.7%	0.79 (0.24, 2.59)
	Asian	3.7%	34.9%	**1.82 (1.03, 3.22)**	18.4%	**2.94 (1.40, 6.17)**	5.4%	2.06 (0.78, 5.45)
	Black	15.0%	23.3%	1.28 (0.78, 2.10)	10.6%	**2.38 (1.18, 4.77)**	5.2%	**3.40 (1.04, 11.09)**
	Other	2.6%	29.9%	1.64 (0.63, 4.26)	18.0%	**3.95 (1.19, 13.15)**	13.3%	**7.73 (1.64, 36.42)**
	White	65.4%	22.0%	Ref.	5.9%	Ref.	2.2%	Ref.
	**Education**							
	HS or less	48.4%	22.7%	1.06 (0.62, 1.84)	8.0%	0.64 (0.29, 1.41)	2.6%	0.70 (0.27, 1.78)
	Some college	34.0%	23.7%	1.10 (0.67, 1.81)	7.2%	0.58 (0.30, 1.12)	2.4%	0.60 (0.12, 0.88)
	College or more	17.6%	25.8%	Ref.	14.3%	Ref.	4.9%	Ref.
	**Income**							
	<$50,000	61.1%	20.2%	**0.63 (0.41, 0.96)**	6.4%	**0.47 (0.26, 0.84)**	1.7%	**0.32 (0.12, 0.88)**
	$50,000+	38.9%	29.2%	Ref.	12.7%	Ref.	4.9%	Ref.
	**Urbanicity**							
	Rural	34.9%	25.9%	0.95 (0.57, 1.59)	8.4%	0.67 (0.33, 1.38)	4.2%	1.16 (0.49, 2.75)
	Small city	9.3%	15.8%	**0.45 (0.41, 0.96)**	5.8%	**0.30 (0.15, 0.58)**	1.0%	**0.23 (0.07, 0.73)**
	Suburban	28.8%	20.6%	**0.58 (0.37, 0.92)**	6.2%	**0.27 (0.14, 0.52)**	2.1%	**0.37 (0.13, 0.99)**
	Small town	14.0%	22.3%	0.72 (0.40, 1.28)	7.3%	0.46 (0.18, 1.14)	0.9%	0.25 (0.04, 1.81)
	Big city	13.0%	31.9%	Ref.	19.7%	Ref.	4.8%	Ref.
**Tobacco products use status**	**Cigarettes**							
	Yes	74.9%	22.9%	1.20 (0.80, 1.81)	8.2%	1.22 (0.69, 2.16)	2.6%	1.54 (0.66, 3.58)
	No	25.1%	25.7%	Ref.	10.8%	Ref.	3.9%	Ref.
	**Electronic vaping products**							
	Yes	30.4%	34.1%	**1.99 (1.29, 3.06)**	17.7%	**2.30 (1.29, 4.09)**	8.7%	**17.04 (4.63, 62.66)**
	No	69.6%	19.0%	Ref.	5.0%	Ref.	0.4%	Ref.
	**Cigars**							
	Yes	20.6%	34.9%	1.38 (0.84, 2.28)	20.3%	1.46 (0.77, 2.77)	6.8%	0.50 (0.20, 1.23)
	No	79.4%	29.7%	Ref.	5.9%	Ref.	2.0%	Ref.
	**Hookah**							
	Yes	11.0%	40.5%	1.72 (0.90, 3.28)	30.3%	**2.78 (1.22, 6.30)**	14.2%	**6.00 (2.27, 15.87)**
	No	89.0%	21.5%	Ref.	6.2%	Ref.	1.6%	Ref.
	**Other combustibles**							
	Yes	15.7%	34.4%	1.22 (0.71, 2.07)	22.4%	1.79 (0.97, 3.31)	10.0%	**3.42 (1.29, 9.05)**
	No	84.3%	21.6%	Ref.	6.4%	Ref.	1.7%	Ref.
	**Smokeless tobacco**							
	Yes	13.8%	29.4%	0.73 (0.43, 1.23)	19.4%	0.89 (0.47, 1.71)	8.1%	0.56 (0.21, 1.50)
	No	86.2%	22.7%	Ref.	7.2%	Ref.	2.1%	Ref.

Boldface indicates statistical significance (*p* < 0.05). Individuals who were unaware of heated tobacco products were categorized as never users when ever use was the outcome, and non-current users when current use was the outcome. The overall column presents distributions of weighted sample characteristics. The percentages under aware, ever use, and current use represent prevalence of the corresponding measure in each sociodemographic and tobacco products use category. Estimates for demographics are adjusted for demographics only. Estimates for tobacco product use status were adjusted for all variables in the table.

**Table 2:** Weighted prevalence of HTP-related beliefs and their associations with HTP use status, 2021 (n = 1582).

Beliefs	Overall	HTP Use Status						
		Non-Susceptible Never (n = 407)	Susceptible Never (n = 976)		Ever-Not-Current (n = 145)		Current (n = 54)	
		% Agree	% Agree	AOR (95% CI)	% Agree	AOR (95% CI)	% Agree	AOR (95% CI)
**IQOS is Less Harmful than Cigarettes**								
Agree	18.3%	3.2%	21.1%	**4.97 (2.46, 10.04)**	48.7%	**7.90 (3.06, 20.38)**	53.9%	**10.52 (3.05, 36.2)**
Don’t know	53.4%	69.4%	50.6%	0.84 (0.53, 1.32)	18.4%	**0.28 (0.11, 0.75)**	20.4%	0.82 (0.23, 2.93)
Disagree	28.3%	27.4%	28.4%	Ref.	32.9%	Ref.	25.7%	Ref.
**IQOS is less addictive than cigarettes**								
Agree	15.0%	2.7%	16.6%	**4.10 (1.81, 9.33)**	38.8%	**5.71 (2.04, 15.97)**	57.1%	**10.18 (2.99, 34.62)**
Don’t know	52.5%	66.9%	50.3%	0.84 (0.54, 1.31)	21.4%	**0.31 (0.13, 0.74)**	15.1%	0.46 (0.12, 1.78)
Disagree	32.5%	30.4%	33.0%	Ref.	39.9%	Ref.	27.8%	Ref.
**IQOS is less harmful than electronic vaping products**								
Agree	14.5%	0.8%	16.3%	**12.43 (5.24, 29.49)**	41.0%	**16.87 (5.33, 53.45)**	62.7%	**68.25 (17.67, 263.67)**
Don’t know	57.4%	73.2%	54.7%	0.78 (0.50, 1.12)	22.9%	**0.28 (0.13, 0.60)**	21.7%	1.15 (0.32, 4.16)
Disagree	28.1%	26.0%	29.1%	Ref.	36.0%	Ref.	15.6%	Ref.
**IQOS is less addictive than electronic vaping products**								
Agree	13.5%	1.5%	14.6%	**4.82 (1.98, 11.77)**	43.2%	**10.65 (3.56, 31.91)**	51.1%	**15.27 (4.49, 51.96)**
Don’t know	54.8%	72.1%	50.8%	**0.64 (0.41, 0.98)**	25.5%	**0.37 (0.16, 0.87)**	22.2%	0.79 (0.26, 2.44)
Disagree	31.7%	26.4%	34.6%	Ref.	31.4%	Ref.	26.7%	Ref.
**Using IQOS occasionally is not harmful**								
Agree	16.3%	2.6%	18.6%	**5.89 (2.69, 12.89)**	41.3%	**8.99 (3.15, 25.67)**	58.5%	**32.84 (10.41, 103.56)**
Don’t know	50.6%	65.9%	47.0%	0.86 (0.56, 1.32)	23.7%	0.46 (0.21, 1.01)	23.3%	1.79 (0.57, 5.62)
Disagree	33.1%	31.5%	34.4%	Ref.	34.9%	Ref.	18.2%	Ref.
**Using IQOS occasionally is not addictive**								
Agree	13.5%	3.8%	14.1%	**2.02 (0.88, 4.60)**	36.2%	**2.75 (0.93, 8.22)**	53.8%	**3.65 (1.05, 12.69)**
Don’t know	49.4%	64.2%	46.9%	0.75 (0.49, 1.14)	17.1%	**0.22 (0.09, 0.54)**	14.3%	0.40 (0.10, 1.57)
Disagree	37.1%	32.0%	39.0%	Ref.	46.7%	Ref.	31.9%	Ref.
**IQOS does not harm bystanders**								
Agree	19.7%	3.8%	24.1%	**4.86 (2.14, 11.05)**	39.1%	**4.77 (1.60, 14.23)**	51.1%	**10.54 (3.27, 33.94)**
Don’t know	52.9%	72.9%	48.0%	0.74 (0.46, 1.18)	19.4%	**0.23 (0.09, 0.57)**	17.6%	0.57 (0.17, 1.84)
Disagree	27.4%	23.3%	27.9%	Ref.	41.5%	Ref.	31.2%	Ref.
**IQOS is socially acceptable**								
Agree	23.0%	4.5%	27.2%	**5.99 (2.66, 13.49)**	53.6%	**7.49 (2.74, 20.51)**	64.1%	**19.02 (5.31, 68.16)**
Don’t know	51.1%	68.2%	48.0%	0.95 (0.59, 1.55)	12.1%	**0.20 (0.09, 0.45)**	21.8%	1.51 (0.42, 5.38)
Disagree	25.8%	27.3%	24.8%	Ref.	34.6%	Ref.	14.0%	Ref.
**IQOS is approved by the FDA to be safe**								
Agree	15.4%	3.00%	17.5%	**3.14 (1.42, 6.92)**	35.8%	**3.50 (1.30, 9.42)**	57.2%	**10.86 (3.05, 38.68)**
Don’t know	62.1%	78.8%	58.2%	0.66 (0.40, 1.10)	33.4%	**0.33 (0.14, 0.75)**	29.5%	0.95 (0.30, 3.07)
Disagree	22.5%	18.2%	24.3%	Ref.	30.8%	Ref.	13.4%	Ref.

Boldface indicates statistical significance (*p* < 0.05). HTP = Heated Tobacco Product. Each belief was modeled separately, adjusting for demographics and tobacco product use statuses.

## Data Availability

Data can be made available upon request.
